# Impedance and Electrically Evoked Compound Action Potential (ECAP) Drop within 24 Hours after Cochlear Implantation

**DOI:** 10.1371/journal.pone.0071929

**Published:** 2013-08-26

**Authors:** Joshua Kuang-Chao Chen, Ann Yi-Chiun Chuang, Georg Mathias Sprinzl, Tao-Hsin Tung, Lieber Po-Hung Li

**Affiliations:** 1 Department of Otolaryngology, Cheng Hsin General Hospital, Taipei, Taiwan; 2 Department of Medical Research and Education, Cheng Hsin General Hospital, Taipei, Taiwan; 3 Faculty of Medicine, School of Medicine, National Yang-Ming University, Taipei, Taiwan; 4 Mackay Memorial Hospital, Taipei, Taiwan; 5 Department of Oto-Rhino-Laryngology, Medical University Innsbruck, Innsbruck, Austria; 6 Integrated Brain Research Laboratory, Department of Medical Research and Education, Taipei Veterans General Hospital, Taipei, Taiwan; University of Salamanca- Institute for Neuroscience of Castille and Leon and Medical School, Spain

## Abstract

Previous animal study revealed that post-implantation electrical detection levels significantly declined within days. The impact of cochlear implant (CI) insertion on human auditory pathway in terms of impedance and electrically evoked compound action potential (ECAP) variation within hours after surgery remains unclear, since at this time frequency mapping can only commence weeks after implantation due to factors associated with wound conditions. The study presented our experiences with regards to initial switch-on within 24 hours, and thus the findings about the milieus inside cochlea within the first few hours after cochlear implantation in terms of impedance/ECAP fluctuations. The charts of fifty-four subjects with profound hearing impairment were studied. A minimal invasive approach was used for cochlear implantation, characterized by a small skin incision (≈2.5 cm) and soft techniques for cochleostomy. Impedance/ECAP was measured intro-operatively and within 24 hours post-operatively. Initial mapping within 24 hours post-operatively was performed in all patients without major complications. Impedance/ECAP became significantly lower measured within 24 hours post-operatively as compared with intra-operatively (p<0.001). There were no differences between pre-operative and post-operative threshold for air-conduction hearing. A significant drop of impedance/ECAP in one day after cochlear implantation was revealed for the first time in human beings. Mechanisms could be related to the restoration of neuronal sensitivity to the electrical stimulation, and/or the interaction between the matrix enveloping the electrodes and the electrical stimulation of the initial switch-on. Less wound pain/swelling and soft techniques both contributed to the success of immediate initial mapping, which implied a stable micro-environment inside the cochlea despite electrodes insertion. Our research invites further studies to correlate initial impedance/ECAP changes with long-term hearing/speech performance.

## Introduction

Previous research revealed little fluctuation of electrically evoked compound action potential (ECAP) thresholds over a period of five to six years after cochlear implantation (CI) [1]. It has been generally accepted that the measurement of CI electrode impedance (and thus ECAP) was not stabilized within the first days or even weeks post-operatively [2], [3]. Mostly this was thought to be due to ongoing physiological processes related to the implantation of a foreign body in the cochlea and the healing from the trauma related to the implant process [4], [5].The impact of cochlear implant insertion on peripheral auditory pathway, however, has never been addressed in terms of impedance and ECAP level variation within the first few hours after operation. The reason resided in the fact that trials to switch on a newly inserted CI device and launch the process of frequency mapping generally could not commence until weeks after cochlear implantation due to factors associated with wound conditions regardless of various surgical approaches currently utilized.

The difficulty in immediate initial mapping arises mainly from the poor compliance of patients caused by postoperative pain. A locally increased impedance of soft tissues due to subcutaneous edema/hematoma following the surgery furthermore hampered the communication between the receiver and electrodes of the CI apparatus. Air bubbles, blood clots, and bone dust that possibly went in the cochlea because of manipulation during operations all added to the intricacy of this procedure. Thus it is obvious that dilemmas raised here could be ascribed at least in part to the surgical procedure itself, including a generally large-scale skin wound and the “hard” technique frequently used for cochleostomy.

Commencement of electrical stimulation within 24 hours after cochlear implantation has long been performed in our department on a routine basis. By means of a modification of the commonly adopted process for CI operation, initial switch-on within 24 hours was made possible. The modified method in turn could promise a better wound condition with respect to having only a tiny amount of local pain/swelling, and very little contamination of perilymph by air bubbles, blood clots and/or bone dust on electrodes insertion.

In the present study, the milieus inside the cochlea within the first few hours after cochlear implantation in terms of impedance/ECAP fluctuations was disclosed for the first time by using the aforementioned minimal invasive approach for CI operation. The charts of fifty-four patients with a profound degree of hearing impairment were reviewed. Impedance and ECAP were evaluated. Effect of gender and age was also addressed.

## Materials

### Ethics Statement

The study conformed to the Declaration of Helsinki and the guidelines of the Institutional Ethics and Research Committee of the Cheng-Hsin General Hospital. Written informed consent was obtained from each participant or their parents with a protocol approved by the Institutional Ethics and Research Committee of the Cheng Hsin General Hospital.

### Subjects (Table 1)

The charts of fifty-four subjects with a profound degree of hearing impairment (twenty-seven males and twenty-seven females; 1∼80 y/o, mean = 16.4) were studied (Table 1). No other neurological deficits were identified. Thirty-two subjects used Freedom (left = 13, right = 20), four subjects used N5 (left = 1, right = 3), and eighteen subjects used Nucleus 24 (left = 5, right = 13)(Cochlear™, Lane Cove NSW, Australia) cochlear implant system.

**Table 1:** General data for all participants.

		Age (yr)			DuH (day)
No	Gender		Side	Device	
1*	M	1	Rt	freedom	5
2*	M	2	Lt	freedom	5
3	M	3	Rt	Nucleus	2
4	M	3	Lt	Nucleus	4
5*	M	3	Rt	Nucleus	7
6*	M	3	Rt	freedom	4
7*	M	3	Rt	freedom	5
8*	M	3	Rt	Nucleus	5
9*	F	3	Lt	Nucleus	5
10	M	4	Rt	freedom	2
11	F	4	Rt	freedom	2
12*	F	4	Rt	Nucleus	4
13*	F	4	Rt	freedom	14
14*	F	4	Rt	freedom	4
15*	F	4	Rt	freedom	4
16	M	5	Rt	freedom	3
17	F	5	Rt	Nucleus	3
18	F	5	Rt	freedom	7
19	F	6	Lt	freedom	3
20	F	8	Lt	freedom	3
21	F	11	Rt	freedom	3
22*	M	11	Rt	Nucleus	4
23	F	12	Lt	Nucleus	3
24*	F	13	Rt	freedom	4
25	M	13	Lt	freedom	2
26*	F	14	Lt	Nucleus	4
27	M	15	Lt	freedom	3
28	M	15	Lt	freedom	3
29*	M	15	Rt	Nucleus	5
30*	F	16	Rt	Nucleus	5
31	F	19	Lt	freedom	3
32	M	20	Rt	Nucleus	4
33	F	20	Lt	freedom	3
34*	F	22	Lt	freedom	10
35*	M	22	Rt	Nucleus	4
36*	M	23	Lt	freedom	4
37*	M	26	Rt	freedom	9
38	F	29	Rt	Nucleus	4
39	M	38	Rt	freedom	4
40	F	44	Lt	freedom	2
41	M	53	Rt	freedom	3
42	F	61	Lt	Nucleus	3
43	M	69	Rt	freedom	3
44	F	74	Rt	freedom	4
45	M	80	Lt	freedom	2
46*	F	8	Rt	Nucleus	4
47*	F	14	Rt	Nucleus	5
48*	F	2	Rt	freedom	2
49	M	4	Lt	freedom	4
50	M	18	Rt	freedom	4
51*	F	1	Rt	N5	5
52*	M	24	Rt	N5	5
53*	F	1	Lt	N5	5
54*	M	2	Rt	N5	5

No, participant number; Age, y/o; Side, side of implantation; Device, type of cochlear implant; DuH, duration of hospitalization (days); *, international patients.

### Surgical procedures (Figure 1)

Under general anesthesia, patients were put in a supine position with their heads turned 45° away from the operated side. The surgical area was well exposed. After draping and sterilization, a 2.5 cm skin incision just anterior to the hair line was made in post-auricular region. An anterior-based skin flap was elevated by entering the areolar tissue plane and dissecting all the way to the level of Henle's spine anteriorly. A posterior-based musculoperiosteal flap for housing the receiver of cochlear implant on skull was then created by entering the subperiosteal space from the incision at a distance of about 2 cm away from the posterior wall of external ear canal. The dissection was done posteriorly from the level of Henle's spine. A well was made only when needed and no sutures were necessary for the fixation, since the tension of the musculoperiosteal flap was enough to secure the implant in position. The channel for the cable, however, was made on a routine basis. To avoid possible tissue injuries, four custom-made hooks were used to expose the cortex of the mastoid bone instead of using a self-retaining instrument (e.g. mastoid retractor). A thin rectangular chip of cortex bone (about 20×10×3 mm in size) was first harvested intact from the mastoid with a chisel, and then a drill was used for mastoidectomy. The size of this chip would be decided on according to the degree of aeration in the mastoid cavity evaluated by pre-operative computed tomography (CT). Electrocautery was seldom used unless it was necessary throughout the whole procedure in order to minimize both pain and swelling caused by burn. In addition, repeated irrigation with normal saline is mandatory, since residual blood clots and/or bone dusts would lead to significant pain and swelling of the wound postoperatively. Soft techniques [6] were employed for the cochleostomy/round window approach after facial nerve was located/well-preserved by using a facial nerve monitor (Medtronics™, Minneapolis, USA) and facial recess was penetrated. Healon (Advanced Medical Optics™, Uppsala, Sweden) was used for the coverage of cochleostomy/round window to prevent possible loss as well as contamination of perilymph by air bubbles, blood clots and/or bone dusts on electrodes insertion. Method of atraumatic insertion including Advance Off-Stylet (AOS; Cochlear™, Lane Cove NSW, Australia) was applied for the introduction of implant into cochlea to prevent excessive trauma of hair cells. The AOS technique has been demonstrated to be atraumatic and is therefore an important element of soft technique [7]. Following electrodes insertion, the thin chip of cortex bone was used as a shielding coverage to shelter the defect of mastoid cavity for the protection of the wire inside. The wound was closed with 3–0 Vicryl (Ethicon™, New Jersey, USA) layer by layer in a primary intention. A pressure mastoid dressing was then applied. The dressing would be removed in the morning of the first postoperative day. After the initial switch-on of newly-implanted cochlear implant and frequency mapping in the afternoon, patients could always be discharged in the evening.

**Figure 1:**
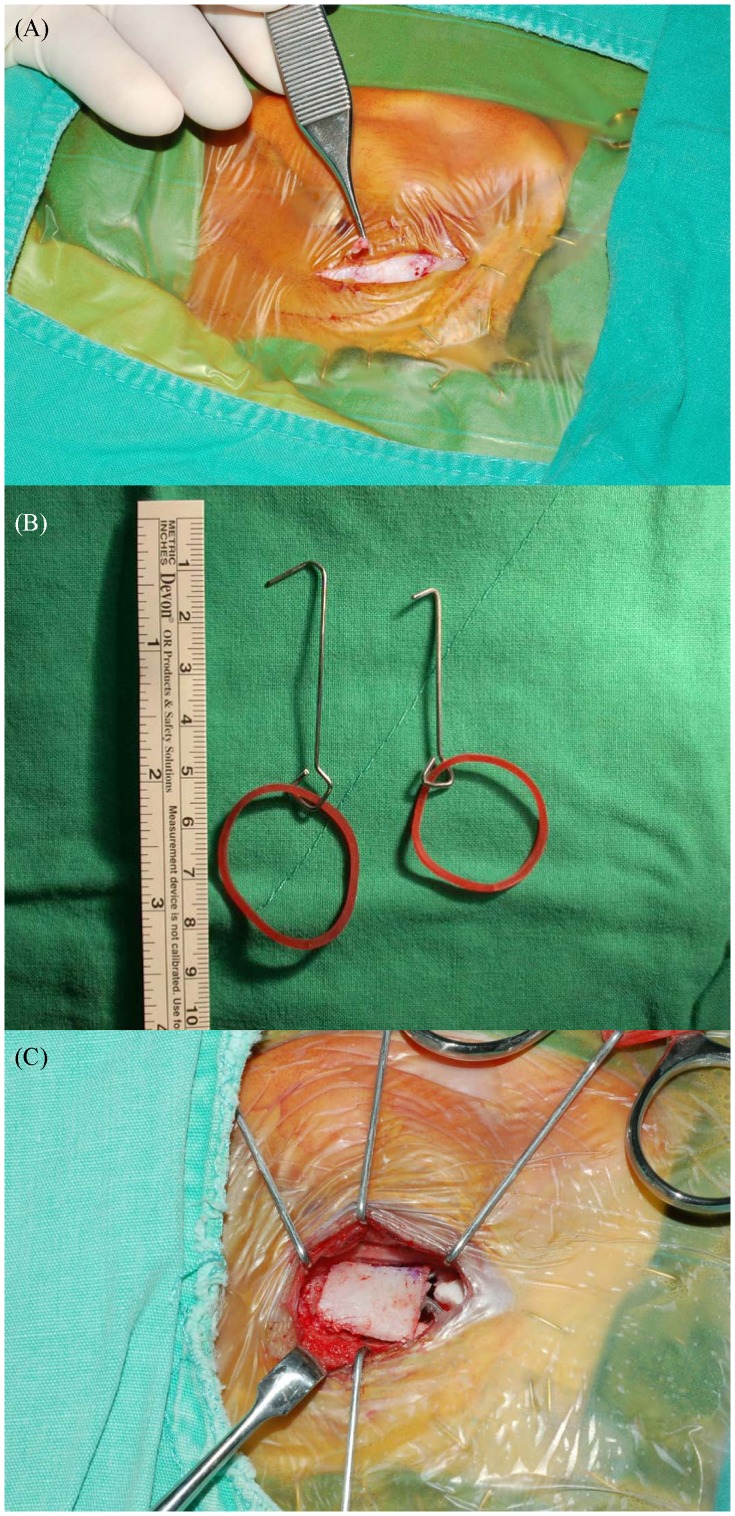
Surgical procedures for a minimal invasive approach of cochlear implantation aiming for immediate initial mapping. *(* ***A*** *)* After draping and sterilization, a 2.5 to 3 cm skin incision was made in the post-auricular region at a distance of about 2 cm away from the posterior wall of external ear canal. *(* ***B*** *)* To avoid possible tissue injuries, four custom-made hooks instead of self-retain instrument (e.g. mastoid retractor) were used to expose the cortex of mastoid bone. *(* ***C*** *)* A thin piece of cortex bone (about 10×10×3 mm in size) harvested before the mastoidectomy was used as a shielding coverage for the protection of the wire inside mastoid cavity.

### Audiometric and Electrophysiological Exam

Impedance and ECAP were recorded intra-operatively as well as within 24 hours after the surgery, respectively. All participants underwent pure tone audiometry (PTA) with sound field exam in an acoustically-shielded room to determine the hearing threshold before (unaided) as well as after (unaided and CI-aided) the operation, using test frequencies between 250 Hz to 8000 Hz. The maximal output of the Grason-Stadler GS61audiometer (Grason-Stadler™, Minnesota, USA; calibrated in line with American National Standards Institute standards) was 120 dB HL for frequencies 250 to 4000 Hz, and 110 dB HL for 8000 Hz. A value of 5 dB was added to the maximal output threshold of the testing frequency where no responses could be found above the limit [8], [9], [10]. Elapsed time after operation for the postoperative PTA exam was about 6 months.

### Data Analysis

Statistical analysis was performed using the software of SAS 8.1 (SAS Institute Inc., Cary, NC, USA). Differences between values of impedance/ECAP measured intra-operatively and within 24 hours post-operatively of channels number 1, 6, 11, 16, and 22 were analyzed using paired t-test. Gender and age (cut-off value at 18 y/o) differences in impedance/ECAP were evaluated using t-test. The threshold for statistical significance was set at P<0.05.

## Results

The hookup between the external transmitting coil and the implanted antenna coil was done in all patients without any discomfort right after the compression dressing was removed, which in turn led to a successful switch-on of cochlear implants and thus initial mapping within 24 hours post-operatively. All patients were well-accustomed to their cochlear implants after initial mapping and usually were willing to keep hooking the transmitting coil up in their daily activities. No pre-operative medication except for one dose of prophylactic antibiotics thirty minutes before the operation was prescribed for all patients. None of the postoperative complications such as hematoma, swelling, infection, or flap failure were noted (Table 2). Only one patient asked for oral analgesics three hours after the surgery due to wound pain.

**Table 2:** Rates of complications.

Complications	No	Rate
Major	0	0
Minor	0	0

No, number; Major complications: hematoma, swelling, infection, flap failure, ect.

**Figure 2:**
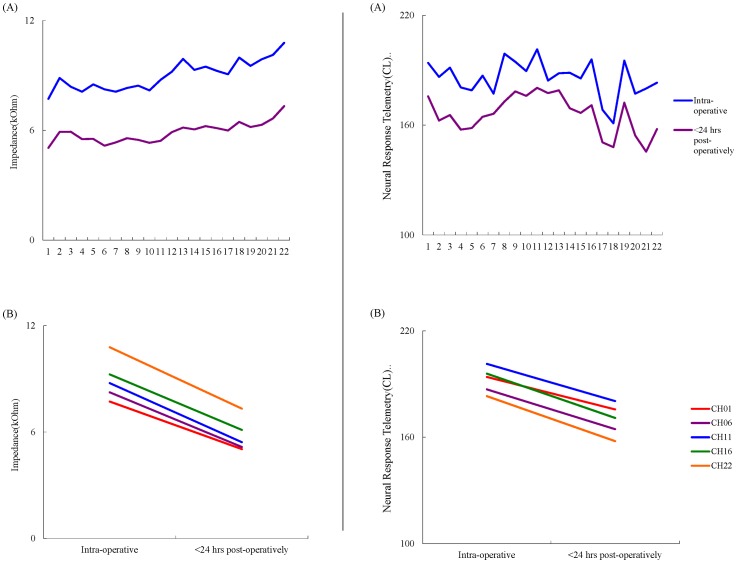
Variations of impedance (left column) and electrical compound action potential (ECAP; right column) measured intra-operatively and within 24 hours post-operatively. *(A)* Impedance and ECAP results of all channels. *(B)* Impedance and ECAP results of selected channels (no 1, 6, 11, 16, and 22). Impedance and ECAP both became significantly lower measured within 24 hours post-operatively than those measured intra-operatively.

Impedance and ECAP both became significantly lower measured within 24 hours post-operatively (mean  = 5.1 kOhm at channel 1, 5.2 kOhm at channel 6, 5.7 kOhm at channel 11, 6.2 kOhm at channel 16, and 8.0 kOhm at channel 22 for impedance; mean  = 182.3 CL at channel 1, 172.0 CL at channel 6, 178.6 CL at channel 11, 162.2 CL at channel 16, and 152.5 CL at channel 22 for ECAP) than those measured intra-operatively (mean  = 8.3 kOhm at channel 1, 8.6 kOhm at channel 6, 9.6 kOhm at channel 11, 10.0 kOhm at channel 16, and 11.7 kOhm at channel 22 for impedance; mean  = 197.3 CL at channel 1, 195.2 CL at channel 6, 200.7 CL at channel 11, 185.3 CL at channel 16, and 175.2 CL at channel 22 for ECAP). There were no gender and age differences between pre-operative and post-operative impedance and ECAP values (Table 3, 4, Table S1 and S2, and Figure 2).

**Table 3:** Impedance for all participants.

			Channel Number		
	1	6	11	16	22
*Intraoperatively*					
m	8.3	8.6	9.6	10.0	11.7
SD	2.8	2.3	2.7	3.0	3.2
*p_1_*	0.79	0.14	0.45	0.25	0.74
*p_2_*	0.52	0.77	0.66	0.91	0.94
*Within 24 hours postoperatively*					
m	5.1	5.2	5.7	6.2	8.0
SD	1.8	1.7	1.6	1.7	2.3
*p_3_*	0.61	0.79	0.72	0.45	0.30
*p_4_*	0.55	0.90	0.90	0.82	0.80
*p_5_*	<0.001	<0.001	<0.001	<0.001	<0.001

Threshold for statistical significance using paired t-test was set at P<0.05. P1, significance of gender difference in impedance measured intraoperatively; P2, significance of gender difference in impedance measured within 24 hours postoperatively; P3, significance of age difference (cut-off value at 18 y/o) in impedance measured intraoperatively; P4, significance of age difference (cut-off value at 18 y/o) in impedance measured within 24 hours postoperatively; P5, significance of difference between impedance measured intraoperatively and that within 24 hours postoperatively.

**Table 4:** Electrically evoked compound action potential (ECAP) for all participants.

			Channel Number		
	1	6	11	16	22
*Intraoperatively*					
m	197.3	195.2	200.7	185.3	175.2
SD	30.6	20.2	14.9	29.2	31.0
*p_1_*	0.65	0.67	0.45	0.54	0.27
*p_2_*	0.08	0.81	0.72	0.69	0.57
*Within 24 hours postoperatively*					
m	182.3	172.0	178.6	162.2	152.5
SD	30.0	21.5	15.2	26.7	24.1
*p_3_*	0.45	0.58	0.51	0.88	0.72
*p_4_*	0.88	0.96	0.72	0.45	0.65
*p_5_*	<0.001	<0.001	<0.001	<0.001	<0.001

Threshold for statistical significance using paired t-test was set at P<0.05. P1, significance of gender difference in electrically evoked compound action potential (ECAP) measured intraoperatively; P2, significance of gender difference in ECAP measured within 24 hours postoperatively; P3, significance of age difference (cut-off value at 18 y/o) in ECAP measured intraoperatively; P4, significance of age difference (cut-off value at 18 y/o) in ECAP measured within 24 hours postoperatively; P5, significance of difference between ECAP measured intraoperatively and that within 24 hours postoperatively.

There were no differences between pre-operative (mean  = 96 dB HL at 500 Hz, 103 dB HL at 1000 Hz, 106 dB HL at 2000 Hz, and 111 dB HL at 4000 Hz) and post-operative (mean  = 112 dB HL at 500 Hz, 118 dB HL at 1000 Hz, 120 dB HL at 2000 Hz, and 121 dB HL at 4000 Hz) threshold for air-conduction hearing (Table 5).

**Table 5:** Hearing for all participants.

		Frequency (Hz)		
	500	1000	2000	4000
*Pre-operative*				
m	96	103	106	111
SD	16.9	15.9	15.5	18.2
*Postoperative*				
m	112	118	120	121
SD	10.4	6.9	6.0	4.4
*p*	0.88	0.88	0.15	0.51

Threshold for statistical significance using pair-t test was set at P<0.05. P, significance of difference between pre-operative and post-operative hearing threshold.

## Discussion

One major and novel finding in this study was the significant drop in the level for impedance/ECAP in less than one day after cochlear implantation. Impedance/ECAP measured within 24 hours post-operatively decreased considerably as compared with those measured intra-operatively in our subjects. Our finding was thus in line with one previous study that post-implantation electrical detection levels significantly declined within days in some animals [11]. Due to the aforementioned difficulty of instant switch-on for cochlear implant inserted via a technique of traditionally large-incision approach, issues regarding the levels of electrical thresholds in the first few hours and/or days following electrodes insertion were dealt with only in animal models. To the best of our knowledge, this is the first research yet disclosing results about diminished impedance/ECAP within 24 hours subsequent to cochlear implantation in human beings.

Mechanism(s) underlying the reduced values of impedance/ECAP within one day after the surgery in our subjects remained unclear. One possibility was the interaction between the matrix enveloping the electrodes and the electrical stimulation of the initial switch-on [12]. It has been revealed that a tissue sheath would be created around electrodes shortly after the insertion [4], [13]. This sheath has been assumed to cause an increase in the impedance/ECAP of the individual electrode contacts. Upon the initial switch-on, however, the electric stimulation could lead to the reorganization or “blow-out (possibly due to bubbles formation)” of the thin and newly-formed tissue sheath [12], [14]. It is thus reasonable to conjecture that the changes in the status of tissue sheath in turn would result in a decrease of the impedance/ECAP, since the grade and continuity of tissue growth surrounding the implant have been shown to be proportionately associated with the increase of the impedance [14], [15].

One more plausible explanation is a spontaneous recovery of micro-environment inside cochlea from that disturbed by electrodes insertion [11], which in turn could lead to a restoration of the original level in impedance and thus ECAP. Histological alterations have been shown to be present within hours after chemical and/or physical trauma to the cochlea in animals [16], [17]. The foreign body reaction combined with other physiological responses within the cochlea, which were thought to be related to the process of drilling around the cochlea/round window niche/cochleostomy [18], would furthermore add to the intricacy of impedance/ECAP measurement. Some of these changes such as swelling of auditory nerve fibers, which could cause a decrease in the sensitivity of neurons to electrical signals, were found to be reversible shortly following the insulting event [19]. The reversibility might hence help to resume the sensitivity of neuronal membranes to the electrical stimulation from cochlear implant, as could be indexed by the post-implantation decrease of electrical thresholds observed in the above-mentioned animal study [11]. In the present study, adverse effects on cochlea resulting from extreme heat produced by the drilling process would mostly be averted by using soft techniques during the course of cochleostomy/round window approach. Healon was then used for the coverage of cochleostomy/round window to prevent possible loss as well as contamination of perilymph by air bubbles and blood clots/bone dusts on electrodes insertion. Furthermore, the skill of “atraumatic” insertion including AOS was applied to avoid excessive trauma of hair cells [7]. The employment of soft techniques and of “atraumatic” method for electrodes insertion could therefore promise a less disturbed micro-environment inside cochlea for the monitoring of impedance/ECAP.

Another possibility to consider could be the involvement of reciprocal interactions, similar to those noted in patients with idiopathic sudden sensorineural hearing loss [20], [21], [22], [23], between central and peripheral auditory pathways induced by the restitution of electrical signals from cochlear implant. It has been revealed that plastic changes in central auditory pathway, probably coupled with negative impacts on neurotransmission/neuromodulation, might be instantly provoked by pathological damages (e.g. electrodes insertion) to the cochlea in animals [24], [25]. The effect of intra-operative electrical stimulation from cochlear implant, on the contrary, could launch a corresponding reorganization for the mending in the central auditory pathway [24], [25] and/or tissue development in peripheral auditory pathway (i.e. the implanted cochlea) [4], [11], [26], [27]. Such reorganization and/or tissue formation would help to avoid further deterioration in the auditory pathways, which sequentially could initiate an improvement in the sensitivity of the whole auditory system as was mirrored by the diminished values of impedance and hence ECAP approximately 24 hours later.

In our study, 27 out of 54 patients came from regions outside the country (i.e. 50%, see Table 1). The strategy of immediate initial switch-on in turn shortened the duration of uncertainty/worry for patients as well as families about surgical outcome, since all of our subjects knew they have had a well-functioning cochlear implant in less than one day after the operation. Patients can always be able to go back to school or rehabilitation center of their hometown two days postoperatively with their “new ears”. Our modified techniques for cochlear implantation thus made possible a nonstop rehabilitation programs peri-operatively for these international implantees. Furthermore, earlier initial switch-on did not imply more frequent visits postoperatively. Due to the good wound condition, patients were only required to come back for routine checkup on 28th post-operative day after discharge.

In summary, a significant drop of impedance and ECAP within 24 hours after cochlear implantation was revealed in the present study. A minimal invasive approach for cochlear implantation made initial mapping and thus the measurement of impedance/ECAP within 24 hours after the surgery possible. Whilst the drop was noted for the first time in human beings within less than one day following electrodes insertion, it seemed to imply that the milieus of the disturbed cochlea could recover within 24 hours postoperatively which in turn could justify the rationale for the instant switch-on of a newly implanted CI device. The strategy of initial switch-on within 24 hours post-operatively also led to benefits such as fewer uncertainty/worry for patients/families about surgical outcomes and nonstop rehabilitation programs. Those benefits were especially important for international patients. Our research invites further studies on a larger group of implanted patients to validate the long-term scenario of impedance/ECAP variations. A longitudinal study is also needed to correlate impedance/ECAP changes at the very beginning of electrodes insertion and hearing/speech performance over time.

## Supporting Information

Table S1 **Impedance for all participants.**
(DOC)

Table S2 **Electrically evoked compound action potential (ECAP) for all participants.**
(DOC)
