# Supplementary material for: Impedance and Electrically Evoked Compound Action Potential (ECAP) Drop within 24 Hours after Cochlear Implantation
Source: PLoS One. 2013 Aug 26;8(8):e71929. doi: 10.1371/journal.pone.0071929 (PMC3753283; doi:10.1371/journal.pone.0071929)
Supplement: Table S1 — Impedance for all participants. (DOC) [file pone.0071929.s001.doc]

**Supplementary information**

**Table S1. Impedance for all participants.**

| **Table S1. Impedance for all participants.** | | | | | |
| --- | --- | --- | --- | --- | --- |
|  |  |  | Channel Number |  |  |
| No | 1 | 6 | 11 | 16 | 22 |
| *Intraoperatively* | | |  |  |  |
| 1 | 4.4 | 7.5 | 7.1 | 5.9 | 13.0 |
| 2 | 9.1 | 9.9 | 10.5 | 12.3 | 17.3 |
| 3 | 4.1 | 6.9 | 13.5 | 10.3 | 10.3 |
| 4 | 14.2 | 5.3 | 3.0 | 5.6 | 9.1 |
| 5 | 9.1 | 9.9 | 7.9 | 9.1 | 9.1 |
| 6 | 4.7 | 4.5 | 7.3 | 7.3 | 5.6 |
| 7 | 9.4 | 9.2 | 11.4 | 10.3 | 18.3 |
| 8 | 13.5 | 12.5 | 13.0 | 14.1 | 10.2 |
| 9 | 4.8 | 6.5 | 8.2 | 8.3 | 13.3 |
| 10 | 4.8 | 5.0 | 10.5 | 11.4 | 15.8 |
| 11 | 10.3 | 4.6 | 9.8 | 8.3 | 8.7 |
| 12 | 7.6 | 6.9 | 8.1 | 14.9 | 10.3 |
| 13 | 6.1 | 7.0 | 7.2 | 7.4 | 9.7 |
| 14 | 5.6 | 7.1 | 8.1 | 6.5 | 8.8 |
| 15 | 8.8 | 6.4 | 9.2 | 9.2 | 11.0 |
| 16 | 6.1 | 11.5 | 8.2 | 9.1 | 14.5 |
| 17 | 12.0 | 9.6 | 11.2 | 12.8 | 17.4 |
| 18 | 10.7 | 13.4 | 15.8 | 14.3 | 15.9 |
| 19 | 9.3 | 7.9 | 9.1 | 7.8 | 10.4 |
| 20 | 13.5 | 9.8 | 11.6 | 12.8 | 14.9 |
| 21 | 6.2 | 7.2 | 7.1 | 6.2 | 12.1 |
| 22 | 8.0 | 7.3 | 6.4 | 6.9 | 11.7 |
| 23 | 10.9 | 10.7 | 13.3 | 12.5 | 14.1 |
| 24 | 7.4 | 6.4 | 9.9 | 8.1 | 11.7 |
| 25 | 6.0 | 7.7 | 7.3 | 9.8 | 10.3 |
| 26 | 5.8 | 8.5 | 7.9 | 8.1 | 7.9 |
| 27 | 6.2 | 7.5 | 11.1 | 5.4 | 9.9 |
| 28 | 10.3 | 11.0 | 13.0 | 11.9 | 16.7 |
| 29 | 7.5 | 11.1 | 9.2 | 8.9 | 9.8 |
| 30 | 5.3 | 7.1 | 11.1 | 9.5 | 13.6 |
| 31 | 9.1 | 7.0 | 7.8 | 6.9 | 9.2 |
| 32 | 5.6 | 8.1 | 7.4 | 9.8 | 11.7 |
| 33 | 5.1 | 6.7 | 4.5 | 4.3 | 4.8 |
| 34 | 13.2 | 14.8 | 13.5 | 12.8 | 14.3 |
| 35 | 6.8 | 8.7 | 5.7 | 7.2 | 6.8 |
| 36 | 8.0 | 9.7 | 11.9 | 12.3 | 14.6 |
| 37 | 9.3 | 8.3 | 7.1 | 11.3 | 13.0 |
| 38 | 4.9 | 6.0 | 7.1 | 10.7 | 9.6 |
| 39 | 5.3 | 5.7 | 8.1 | 8.7 | 6.8 |
| 40 | 7.9 | 6.9 | 9.3 | 10.2 | 11.1 |
| 41 | 7.9 | 10.4 | 9.1 | 12.0 | 9.4 |
| 42 | 9.5 | 6.2 | 7.6 | 6.2 | 6.4 |
| 43 | 6.1 | 7.1 | 7.3 | 6.8 | 13.6 |
| 44 | 7.7 | 9.7 | 14.4 | 12.8 | 13.2 |
| 45 | 12.9 | 9.6 | 13.4 | 19.4 | 13.6 |
| 46 | 8.1 | 9.3 | 10.6 | 10.4 | 8.2 |
| 47 | 12.8 | 12.5 | 14.4 | 10.7 | 14.1 |
| 48 | 14.5 | 13.4 | 13.5 | 15.4 | 16.6 |
| 49 | 7.2 | 11.0 | 10.3 | 9.7 | 12.5 |
| 50 | 7.2 | 8.1 | 8.4 | 7.1 | 9.5 |
| 51 | 9.8 | 10.6 | 9.7 | 14.0 | 12.2 |
| 52 | 10.3 | 9.4 | 10.0 | 10.7 | 9.6 |
| 53 | 10.1 | 8.2 | 11.5 | 14.3 | 16.1 |
| 54 | 9.4 | 9.7 | 7.6 | 11.0 | 13.0 |
| m | 8.3 | 8.6 | 9.6 | 10.0 | 11.7 |
| SD | 2.8 | 2.3 | 2.7 | 3.0 | 3.2 |
| *Within 24 hours postoperatively* | | | | |  |
| 1 | 3.7 | 4.5 | 4.9 | 4.2 | 8.1 |
| 2 | 3.8 | 4.6 | 6.4 | 5.9 | 9.1 |
| 3 | 3.4 | 4.2 | 8.0 | 7.4 | 7.8 |
| 4 | 2.3 | 1.7 | 1.9 | 4.0 | 7.2 |
| 5 | 5.4 | 4.8 | 4.2 | 5.7 | 4.9 |
| 6 | 3.0 | 3.3 | 4.3 | 5.2 | 4.9 |
| 7 | 4.9 | 4.3 | 5.6 | 6.1 | 13.6 |
| 8 | 7.0 | 5.2 | 5.1 | 6.5 | 5.7 |
| 9 | 3.7 | 5.3 | 4.4 | 5.6 | 9.9 |
| 10 | 4.8 | 4.4 | 7.9 | 8.9 | 13.0 |
| 11 | 3.5 | 3.5 | 5.4 | 7.0 | 6.4 |
| 12 | 3.5 | 4.2 | 5.7 | 10.5 | 6.0 |
| 13 | 3.6 | 3.7 | 5.2 | 4.5 | 7.0 |
| 14 | 3.5 | 4.1 | 6.3 | 6.3 | 5.2 |
| 15 | 4.9 | 3.9 | 5.6 | 5.9 | 9.2 |
| 16 | 3.9 | 5.2 | 4.5 | 5.7 | 9.7 |
| 17 | 5.0 | 4.2 | 5.5 | 4.9 | 12.7 |
| 18 | 4.6 | 6.4 | 8.1 | 9.3 | 11.7 |
| 19 | 3.6 | 3.3 | 4.4 | 4.4 | 5.2 |
| 20 | 4.2 | 4.0 | 4.9 | 5.5 | 8.3 |
| 21 | 5.1 | 4.3 | 4.6 | 4.8 | 7.9 |
| 22 | 4.6 | 4.5 | 4.1 | 4.2 | 8.1 |
| 23 | 9.6 | 9.8 | 8.2 | 7.4 | 10.0 |
| 24 | 4.5 | 3.9 | 5.7 | 5.3 | 8.4 |
| 25 | 4.4 | 4.6 | 4.4 | 6.1 | 7.7 |
| 26 | 4.6 | 4.5 | 4.8 | 5.5 | 6.1 |
| 27 | 4.9 | 9.1 | 7.8 | 4.0 | 7.6 |
| 28 | 7.4 | 5.8 | 8.7 | 7.1 | 10.4 |
| 29 | 4.0 | 4.4 | 4.2 | 5.0 | 5.7 |
| 30 | 4.1 | 4.9 | 7.2 | 6.2 | 9.2 |
| 31 | 4.3 | 3.7 | 4.3 | 3.9 | 5.3 |
| 32 | 4.2 | 5.8 | 5.3 | 7.5 | 7.9 |
| 33 | 3.8 | 6.2 | 3.6 | 3.6 | 4.3 |
| 34 | 6.6 | 5.7 | 5.4 | 5.7 | 9.8 |
| 35 | 4.0 | 5.0 | 4.7 | 5.2 | 4.9 |
| 36 | 4.8 | 5.6 | 8.5 | 7.6 | 9.7 |
| 37 | 5.0 | 4.9 | 5.4 | 6.6 | 9.0 |
| 38 | 3.2 | 3.6 | 3.8 | 6.0 | 6.0 |
| 39 | 3.5 | 4.3 | 5.0 | 6.7 | 6.0 |
| 40 | 5.8 | 5.9 | 4.8 | 5.5 | 7.2 |
| 41 | 9.6 | 9.5 | 8.9 | 8.8 | 9.6 |
| 42 | 7.3 | 5.1 | 5.1 | 5.9 | 5.5 |
| 43 | 8.2 | 7.8 | 4.3 | 4.9 | 10.6 |
| 44 | 10.0 | 9.6 | 7.4 | 6.9 | 5.5 |
| 45 | 9.1 | 5.4 | 6.5 | 12.3 | 7.9 |
| 46 | 4.2 | 4.8 | 5.3 | 5.1 | 5.9 |
| 47 | 6.2 | 5.6 | 9.0 | 6.0 | 9.1 |
| 48 | 8.4 | 7.7 | 8.9 | 8.1 | 12.1 |
| 49 | 4.3 | 8.0 | 6.9 | 6.7 | 9.2 |
| 50 | 5.3 | 4.6 | 4.9 | 4.7 | 7.4 |
| 51 | 4.1 | 4.3 | 4.3 | 5.2 | 6.0 |
| 52 | 6.9 | 5.2 | 6.0 | 5.4 | 7.2 |
| 53 | 6.0 | 5.0 | 5.8 | 9.6 | 10.0 |
| 54 | 4.4 | 4.6 | 4.0 | 7.4 | 8.1 |
| m | 5.1 | 5.2 | 5.7 | 6.2 | 8.0 |
| SD | 1.8 | 1.7 | 1.6 | 1.7 | 2.3 |
| *p* | <0.001 | <0.001 | <0.001 | <0.001 | <0.001 |
|  |  |  |  |  |  |
| Threshold for statistical significance using paired t-test was set at P < 0.05. P, significance of difference between impedance measured intraoperatively and that within 24 hours postoperatively. | | | | | |
