# Supplementary material for: Impedance and Electrically Evoked Compound Action Potential (ECAP) Drop within 24 Hours after Cochlear Implantation
Source: PLoS One. 2013 Aug 26;8(8):e71929. doi: 10.1371/journal.pone.0071929 (PMC3753283; doi:10.1371/journal.pone.0071929)
Supplement: Table S2 — Electrically evoked compound action potential (ECAP) for all participants. (DOC) [file pone.0071929.s002.doc]

**Supplementary information**

**Table S2. Electrically evoked compound action potential (ECAP) for all participants.**

| **Table S2. NRT for all participants.** | | | | |  |
| --- | --- | --- | --- | --- | --- |
|  |  |  | Channel Number |  |  |
| No | 1 | 6 | 11 | 16 | 22 |
| *Intraoperatively* | | |  |  |  |
| 1 | ─ | 224 | ─ | 185 | 168 |
| 2 | 207 | 207 | 192 | ─ | 177 |
| 3 | 225 | 194 | 213 | 218 | 207 |
| 4 | ─ | 155 | 209 | 170 | 47 |
| 5 | 197 | 188 | 186 | 170 | 174 |
| 6 | 214 | 165 | 205 | 172 | 154 |
| 7 | 148 | 177 | 215 | 197 | 159 |
| 8 | 204 | 183 | 198 | 177 | 192 |
| 9 | 150 | 168 | 201 | 72 | 183 |
| 10 | 144 | 198 | 198 | 177 | 132 |
| 11 | 235 | 187 | 199 | 178 | 144 |
| 12 | 237 | 174 | 189 | 215 | 234 |
| 13 | 169 | 191 | 199 | 194 | 114 |
| 14 | 193 | 184 | 207 | 206 | 186 |
| 15 | 169 | 182 | 204 | 186 | 186 |
| 16 | 230 | 243 | 238 | 232 | 222 |
| 17 | 204 | 192 | 204 | 186 | 138 |
| 18 | 212 | 201 | 184 | 168 | 157 |
| 19 | 231 | 215 | 150 | 195 | 195 |
| 20 | ─ | 222 | 219 | 221 | ─ |
| 21 | 142 | 236 | ─ | ─ | ─ |
| 22 | 222 | 195 | 195 | 162 | 182 |
| 23 | 227 | 221 | 203 | 188 | 165 |
| 24 | 162 | 180 | 186 | 171 | 156 |
| 25 | 129 | 201 | 204 | 195 | ─ |
| 26 | 225 | ─ | ─ | ─ | ─ |
| 27 | 185 | 200 | 215 | 185 | 176 |
| 28 | 138 | 204 | 192 | 198 | 195 |
| 29 | 207 | 207 | 210 | 153 | 195 |
| 30 | 159 | 147 | 198 | 174 | 174 |
| 31 | 201 | 202 | 208 | 169 | 180 |
| 32 | 238 | ─ | ─ | ─ | 159 |
| 33 | 177 | 171 | 186 | 159 | ─ |
| 34 | 231 | 239 | 226 | 211 | ─ |
| 35 | 213 | 186 | 201 | 216 | 200 |
| 36 | 236 | 211 | 187 | 196 | 208 |
| 37 | 187 | 185 | 212 | 201 | 192 |
| 38 | ─ | 206 | 229 | 210 | 204 |
| 39 | 231 | 206 | 207 | 208 | 186 |
| 40 | 198 | 198 | 222 | 210 | 192 |
| 41 | 189 | 159 | 177 | 201 | 189 |
| 42 | 192 | 189 | 183 | 87 | 120 |
| 43 | 210 | 198 | 204 | 198 | 186 |
| 44 | 189 | 189 | 189 | 165 | 177 |
| 45 | 156 | 183 | 179 | 218 | ─ |
| 46 | 221 | 179 | 203 | ─ | 185 |
| 47 | 237 | 210 | ─ | 209 | 213 |
| 48 | 203 | 203 | 212 | 181 | 173 |
| 49 | 192 | 183 | 189 | 177 | 165 |
| 50 | 174 | 201 | 198 | 180 | 168 |
| 51 | ─ | 224 | 205 | 207 | 201 |
| 52 | 232 | 191 | 197 | 176 | 162 |
| 53 | 180 | 207 | 213 | 201 | 195 |
| 54 | 216 | 188 | 195 | 154 | 168 |
| m | 197.3 | 195.2 | 200.7 | 185.3 | 175.2 |
| SD | 30.6 | 20.2 | 14.9 | 29.2 | 31.0 |
| *Within 24 hours postoperatively* | | | | |  |
| 1 | ─ | 213 | 198 | 171 | 156 |
| 2 | 169 | 190 | 175 | 175 | 166 |
| 3 | 226 | 181 | ─ | 217 | 178 |
| 4 | ─ | ─ | ─ | 165 | 69 |
| 5 | 184 | 169 | 181 | 160 | ─ |
| 6 | 216 | 168 | 189 | 162 | 151 |
| 7 | 127 | 161 | 197 | 176 | 159 |
| 8 | ─ | ─ | ─ | ─ | ─ |
| 9 | 144 | 132 | 192 | 93 | 110 |
| 10 | ─ | ─ | ─ | ─ | 141 |
| 11 | 196 | 172 | 178 | 154 | 127 |
| 12 | 227 | 176 | 179 | 194 | 158 |
| 13 | 146 | 179 | 188 | 179 | 108 |
| 14 | 183 | 168 | 189 | 156 | 165 |
| 15 | 179 | 167 | 170 | 158 | 161 |
| 16 | 238 | ─ | ─ | ─ | 199 |
| 17 | 173 | 161 | 176 | 161 | 131 |
| 18 | 182 | 173 | 158 | 137 | 139 |
| 19 | 223 | 199 | 187 | 172 | 184 |
| 20 | 225 | 204 | 198 | ─ | ─ |
| 21 | 125 | 191 | 176 | 164 | 170 |
| 22 | 196 | 172 | 172 | 130 | 146 |
| 23 | 173 | 176 | 164 | 149 | 133 |
| 24 | 160 | 160 | 145 | 145 | 145 |
| 25 | 147 | 174 | 183 | 177 | 171 |
| 26 | 197 | 195 | 182 | 155 | 146 |
| 27 | 171 | 185 | 191 | 173 | 165 |
| 28 | 159 | 189 | 192 | 183 | 174 |
| 29 | 150 | 177 | 177 | 186 | 174 |
| 30 | 158 | 96 | 183 | 162 | 153 |
| 31 | 176 | 179 | 185 | 169 | 152 |
| 32 | 207 | ─ | ─ | ─ | ─ |
| 33 | 166 | 163 | 169 | 139 | 174 |
| 34 | 189 | 198 | 189 | 180 | 180 |
| 35 | 192 | 147 | 192 | 183 |  |
| 36 | 213 | 171 | 153 | 129 | 111 |
| 37 | 165 | 162 | 183 | 174 | 165 |
| 38 | 229 | 171 | 175 | 181 | 172 |
| 39 | 209 | 194 | 200 | 188 | 170 |
| 40 | 172 | 175 | 193 | 178 | 160 |
| 41 | 138 | 168 | 123 | 144 | 123 |
| 42 | 176 | 164 | 158 | 80 | 109 |
| 43 | 180 | 147 | 174 | 150 | 141 |
| 44 | 138 | 137 | 171 | 96 | 144 |
| 45 | 145 | 175 | 184 | 196 | 142 |
| 46 | 213 | 150 | 192 | 174 | 159 |
| 47 | 207 | 198 | 192 | 177 | 138 |
| 48 | 194 | 181 | 193 | 166 | 158 |
| 49 | 166 | 130 | 166 | 154 | 147 |
| 50 | 144 | 174 | 177 | 153 | 149 |
| 51 | ─ | ─ | 182 | ─ | 176 |
| 52 | 184 | 160 | 166 | 148 | 121 |
| 53 | 190 | 190 | 195 | 185 | 175 |
| 54 | 240 | 205 | 200 | 210 | 150 |
| m | 182.3 | 172.0 | 178.6 | 162.2 | 152.5 |
| SD | 30.0 | 21.5 | 15.2 | 26.7 | 24.1 |
| *p* | <0.001 | <0.001 | <0.001 | <0.001 | <0.001 |
| Threshold for statistical significance using paired t-test was set at P < 0.05. P, significance of difference between NRT measured intraoperatively and that within 24 hours postoperatively. | | | | | |
